# Comparison of Systemic Treatments for Metastatic Castration-Resistant Prostate Cancer After Docetaxel Failure: A Systematic Review and Network Meta-analysis

**DOI:** 10.3389/fphar.2021.789319

**Published:** 2022-01-18

**Authors:** Junru Chen, Yaowen Zhang, Xingming Zhang, Jinge Zhao, Yuchao Ni, Sha Zhu, Ben He, Jindong Dai, Zhipeng Wang, Zilin Wang, Jiayu Liang, Xudong Zhu, Pengfei Shen, Hao Zeng, Guangxi Sun

**Affiliations:** Department of Urology, Institute of Urology, West China Hospital, Sichuan University, Chengdu, China

**Keywords:** metastatic castration-resistant prostate cancer, network meta-analysis, docetaxel (DOC), enzalutamide (ENZ), abirateron, radium-223 (Ra) 223

## Abstract

**Background:** Lacking head-to-head trial, the optimal treatment for patients with metastatic castration-resistant prostate cancer (mCRPC) after docetaxel failure is unclear. This study is to compare the efficacy and safety of systemic treatments in patients who progressed after docetaxel to aid clinical decision-making.

**Methods:** Databases including MEDLINE, EMBASE, and the Cochrane Library were searched from inception to June 15th, 2021. The outcomes of interest include overall survival (OS), biochemical progression-free survival (bPFS), and serious adverse events (SAEs). The Cochrane risk of bias tools were used to assess study quality. Indirect comparisons of competing treatments were performed via Bayesian network meta-analysis.

**Results:** Five trials with 3,862 patients comparing four treatments (abiraterone, enzalutamide, cabazitaxel, and radium-223) were identified. All the four treatments were associated with improved OS and bPFS relative to best supportive care. Among them, enzalutamide (hazard ratio [HR] = 0.58, 95% credible interval [Crl]: 0.49–0.69) had the highest probability of ranking first in terms of OS, followed by cabazitaxel (HR = 0.70, 95% Crl: 0.59–0.83), radium-223 (HR = 0.71, 95% Crl: 0.56–0.90) and abiraterone (HR = 0.73, 95% Crl: 0.63–0.84). Similarly, enzalutamide (HR = 0.25, 95% Crl: 0.20–0.31) showed the greatest improvement of bPFS, followed by abiraterone (HR = 0.60, 95% Crl: 0.51–0.71) and cabazitaxel (HR = 0.75, 95% Crl: 0.63–0.89). In terms of safety, treatments ranked from the safest to the least safe were radium-223 (OR = 0.58, 95% Crl: 0.20–1.68), enzalutamide (OR = 0.80, 95% Crl: 0.28–2.29), abiraterone (OR = 0.94, 95% Crl: 0.39–2.27) and cabazitaxel (OR = 2.50, 95% Crl: 0.84–7.44).

**Conclusion:** For patients with mCRPC who progressed after docetaxel, enzalutamide may offer the most significant survival benefits and satisfying safety. Cabazitaxel is effective in post-docetaxel settings but associated with a high risk of SAEs. Although network meta-analysis provides indirect comparisons and ranking probabilities, the results should be treated with caution as it cannot replace randomized direct comparison.

**Systematic Review Registration:** https://www.crd.york.ac.uk/prospero/display_record.php?ID=CRD42020223040, identifier CRD42020223040.

## 1 Introduction

Prostate cancer (PCa) is the most common malignancy in men in America accounting for 26% of cancer diagnoses (Siegel et al., 2021). Although most patients with metastatic PCa are initially hormone-sensitive and controlled by androgen-deprivation therapy (ADT), diseases progression is inevitable and patients eventually develop castration-resistant diseases (Galletti et al., 2017). Metastatic castration-resistant prostate cancer (mCRPC) is highly aggressive with a median survival ranging from 17.5 to 34.7 months (Petrylak et al., 2004; Berthold et al., 2008; Beer et al., 2014; Ryan et al., 2015). Docetaxel combined with prednisone is the first to show survival benefits in patients with mCRPC and remains one of the standard first-line treatments for this setting (Cornford et al., 2021). However, most patients receiving docetaxel progress within 1 year (Petrylak et al., 2004; Berthold et al., 2008). Based on survival improvements compared to best supportive care, several regimens including abiraterone, enzalutamide, cabazitaxel, and radium-223 are recommended after docetaxel treatment failure (de Bono et al., 2010; de Bono et al., 2011; Fizazi et al., 2012; Scher et al., 2012; Parker et al., 2013; Sun et al., 2016).

Due to the lack of head-to-head trials comparing these drugs, the optimal treatment for patients with mCRPC who progress after docetaxel is unclear. As such, the current guidelines do not recommend one treatment over the others (Cornford et al., 2021; J Natl Compr Canc Netw, 2021). One preliminary analysis has attempted to compare these active treatments indirectly but was limited by a few number of included studies and the exclusion of radium-223 (Fryzek et al., 2018). In addition, the costs and treatment courses of these drugs vary widely (Pollard et al., 2017). This study aimed to compare the efficacy and safety of systemic treatments for mCRPC after upfront docetaxel failure to assist clinical practice.

## 2. Materials and Methods

### 2.1 Search Strategy and Eligibility Criteria

The protocol of this study was developed following Preferred Reporting Items for Systematic Reviews and Meta-analyses (PRISMA) reporting guideline (Tricco et al., 2018) and prospectively registered in PROSPERO (CRD42020223040). Bibliographic databases including MEDLINE (OVID interface), EMBASE (OVID interface), and the Cochrane Central Register of Controlled Trials were searched from inception to June 15th, 2021. The full search strategy was available in the protocol. The eligibility criteria included: 1) randomized controlled trials (RCTs) or cohorts; 2) patients who received first-line docetaxel for mCRPC and progressed; 3) interventions of interest were abiraterone, enzalutamide, cabazitaxel and radium-223; 4) comparators of interest were best supportive care (BSC) or active drugs; 5) studies reporting survival and safety outcomes. Prednisone alone and prednisone plus mitoxantrone were both regarded as BSC as a recent study showed similar survival outcomes for these two treatments (Green et al., 2015). Reviews, case reports, cohorts, study protocols, abstracts, and dose-escalation trials were excluded.

### 2.2 Study Selection and Data Extraction

Two investigators (JRC and YWZ) screened the titles and abstracts independently using Endnote X9. Full texts of potentially eligible studies were further evaluated to identify the final included studies. Data extraction was performed by two investigators (JRC and YWZ) and double-checked. Disagreements were reconciled by discussion or a third investigator (XMZ). The following data were extracted: study design, recruitment period, follow-up time, sample size, interventions, baseline characteristics, efficacy, and safety outcomes. For trials with multiple publications, the most recent data were extracted. The primary efficacy outcome of interest was overall survival (OS), defined as the time from randomization to death due to any cause. The secondary efficacy outcome was biochemical progression-free survival (bPFS), defined as the time from randomization to prostate-specific antigen (PSA) progression or death due to any cause, whichever occurred first. The safety outcome of interest was any serious adverse event (SAE).

### 2.3 Risk of Bias Assessment

Two investigators (JRC and YWZ) assessed the risk of bias of included studies independently. Disagreements were resolved through consensus. The following five domains were evaluated for RCTs according to the Cochrane framework (Sterne et al., 2019): randomization process, deviation from intended interventions, missing outcome data, measurement of the outcome, selection of reported results. The overall risk of bias of a trial was determined by the worst risk of bias in any of the domains. If multiple concerns were raised for one trial, it was judged as at high risk of bias overall.

### 2.4 Statistical Analysis

We performed the indirect comparisons using the Bayesian framework as recommended by the National Institute for Health and Care Excellence (NICE) of the United Kingdom (Shim et al., 2019). For survival outcomes, hazard ratios (HRs) from individual trials were used to estimate overall HRs and corresponding 95% credible intervals (CrIs). The ALSYMPCA trial included patients with and without previous use of docetaxel, and only data from patients who had received docetaxel were used for analysis. For safety outcomes, the incidence of SAE in each treatment arm was used to estimate the overall odds ratios (ORs) with 95% CrIs. We fitted the consistency model and evaluated heterogeneity using *I* ^*2*^ statistic. A fixed-effect model was used if *I* ^*2*^ > 50%. lMarkov chain Monte Carlo (MCMC) algorithms were applied to estimate treatment effects with 100,000 samples after a 10,000-sample burn-in. Treatment ranking probability was assessed via the surface under the cumulative ranking curve (SUCRA). SUCRA ranges from 0 to 1, with score 1 being the best (Salanti et al., 2011). Subgroup analyses were performed based on patient age, ECOG score, and the absence of visceral metastasis. All analyses were performed using the gemtc and rjags packages within R program.

## 3 Results

### 3.1 Characteristics of Include Studies

**FIGURE 1:**
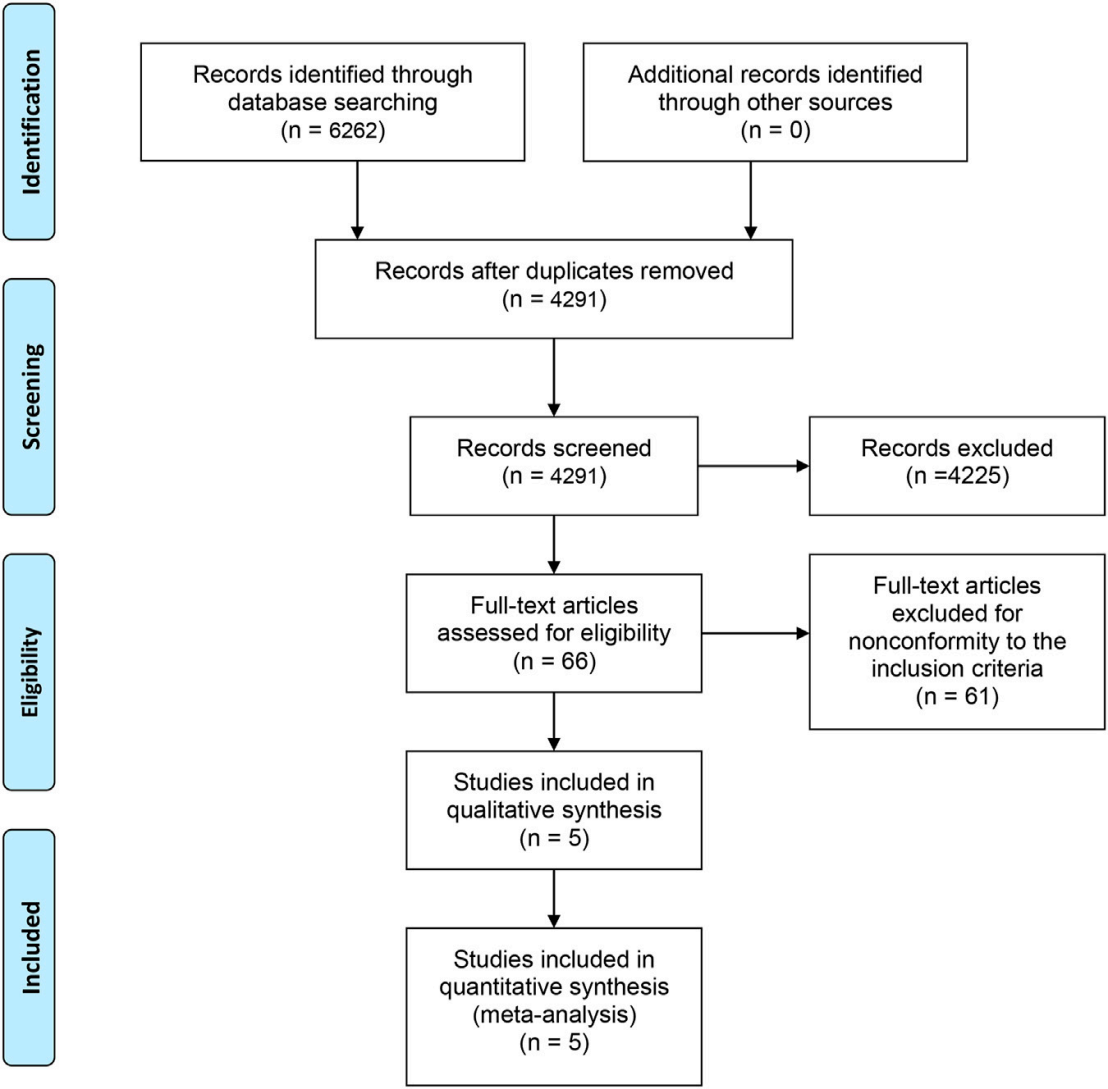
PRISMA flowchart of study selection.

**FIGURE 2:**
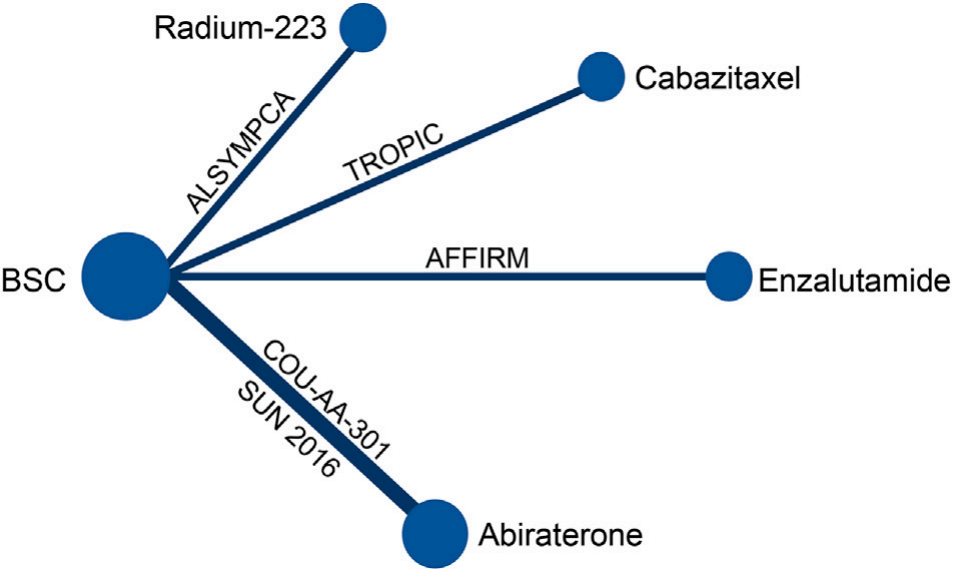
Network plot of treatment comparisons.

A total of 6,262 studies were identified and screened via titles and abstracts. The flowchart showing the selection process is presented in Figure 1. Five trials involving 3,862 patients met our eligibility criteria and were included in this systematic review (de Bono et al., 2010; de Bono et al., 2011; Fizazi et al., 2012; Scher et al., 2012; Parker et al., 2013; Sun et al., 2016). All the included trials were multicenter phase 3 RCTs. The median sample size was 755 (range:214–1,199). The median follow-up duration was 13.7 months (range: 12.8–20.8). The network plot of treatment comparisons is shown in Figure 2. Two trials compared abiraterone + prednisone with prednisone + placebo (de Bono et al., 2011; Fizazi et al., 2012; Sun et al., 2016), one compared enzalutamide with placebo with no requirement of glucocorticoids (Scher et al., 2012), one compared cabazitaxel + prednisone with prednisone + mitoxantrone (de Bono et al., 2010), and one compared radium-223 with placebo (Parker et al., 2013). The ALSYMPCA trial only included patients with bone metastases and no visceral metastases (Parker et al., 2013). The characteristics of included trials and patients are summarized in Tables 1, 2, respectively.

**TABLE 1:** Characteristics of included trials.

Trial	Study design	Recruitment period	Median follow-up (month)	Interventions	Sample size
COU-AA-301	Phase 3, multicenter, double-blind, RCT	May 2008 to Jul 2009	20.2	Abiraterone + prednisone vs. placebo + prednisone	797 vs. 398
Sun et al. (2016)	Phase 3, multicenter, double-blind, RCT	Aug 2012 to Nov 2013	12.9	Abiraterone + prednisone vs. placebo + prednisone	143 vs. 71
AFFIRM	Phase 3, multicenter, double-blind, RCT	Sep 2009 to Nov 2010	14.4	Enzalutamide vs. placebo	800 vs. 399
TROPIC	Phase 3, multicenter, open-label, RCT	Jan 2007 to Oct 2008	12.8	Cabazitaxel + prednisone vs. mitoxantrone + prednisone	378 vs. 377
ALSYMPCA	Phase 3, multicenter, open-label, RCT	Jun 2008 to Feb 2011	NA	Radium-223 vs. placebo	325^a^ vs. 174^a^

Abbreviations: RCT, randomized controlled trial; NA, not available.

a Data of patients with previous docetaxel use.

**TABLE 2:** Baseline characteristics of included patients.

Characteristic	COU-AA-301		Sun et al. (2016)		AFFIRM		TROPIC		ALSYMPCA^a^	
	Abi	Placebo	Abi	Placebo	Enza	Placebo	Caba	Placebo	Ra-223	Placebo
Median age (years)	69	69	Mean: 68.2	Mean: 67.7	69	69	68	67	71	71
Median PSA (ng/ml)	128.8	137.7	Mean: 800.0	Mean: 527.8	107.7	128.3	143.9	127.5	146	173
Gleason score<8	49%	46%	36%	28%	50%	48%	NA	NA	NA	NA
Gleason score ≥8	51%	54%	64%	72%	50%	52%	NA	NA	NA	NA
Bone metastasis	89%	90%	95%	94%	92%	92%	80%	87%	100%	100%
Lung metastasis	13%	11%	8%	13%	15%	15%	Visceral: 25%	Visceral: 25%	0%	0%
Liver metastasis	11%	8%	4%	3%	12%	9%			0%	0%
ECOG score<2	90%	89%	92%	93%	91%	92%	93%	91%	87%	87%
ECOG score≥2	10%	11%	8%	7%	9%	8%	7%	9%	13%	13%

Abbreviations: Abi, abiraterone; Caba, cabazitaxel; Enza: enzalutamide; NA, not available; Ra-223, radium-223.

a Data of overall population.

### 3.2 Risk of Bias

As shown in Table 3, the overall risk of bias was low in three trials (COU-AA-301, Sun et al., 2016 and AFFIRM) (de Bono et al., 2011; Scher et al., 2012; Sun et al., 2016), but some concerns have been raised for the other two trials (TROPIC and ALSYMPCA) (de Bono et al., 2010; Parker et al., 2013). Specifically, deviations from intended interventions might be a concern of bias for the TROPIC trial (de Bono et al., 2010), as patients and treating physicians were not masked to the treatment allocation and there was no information on whether there were deviations from intended intervention because of the trial context. There were concerns of bias regarding measurement of the outcome for the TROPIC and ALSYMPCA trial (de Bono et al., 2010; Parker et al., 2013). In these trials, the outcome assessors were unblinded to intervention status, which might influence the outcome assessment. Additionally, the selection of the reported result raised concerns for the ALSYMPCA trial (Parker et al., 2013), as the protocol was finalized after interim analysis and unblinded outcome data were available for analysis.

**TABLE 3:** Risk of bias of included trials.

Trial	Randomisation process	Deviations from intended interventions	Missing outcome data	Measurement of the outcome	Selection of the reported result	Overall bias
COU-AA-301	Low	Low	Low	Low	Low	Low
Sun et al. (2016)	Low	Low	Low	Low	Low	Low
AFFIRM	Low	Low	Low	Low	Low	Low
TROPIC	Low	Some concerns	Low	Some concerns	Low	Some concerns
ALSYMPCA	Low	Low	Low	Some concerns	Some concerns	Some concerns

### 3.3 Efficacy Outcomes

**FIGURE 3:**
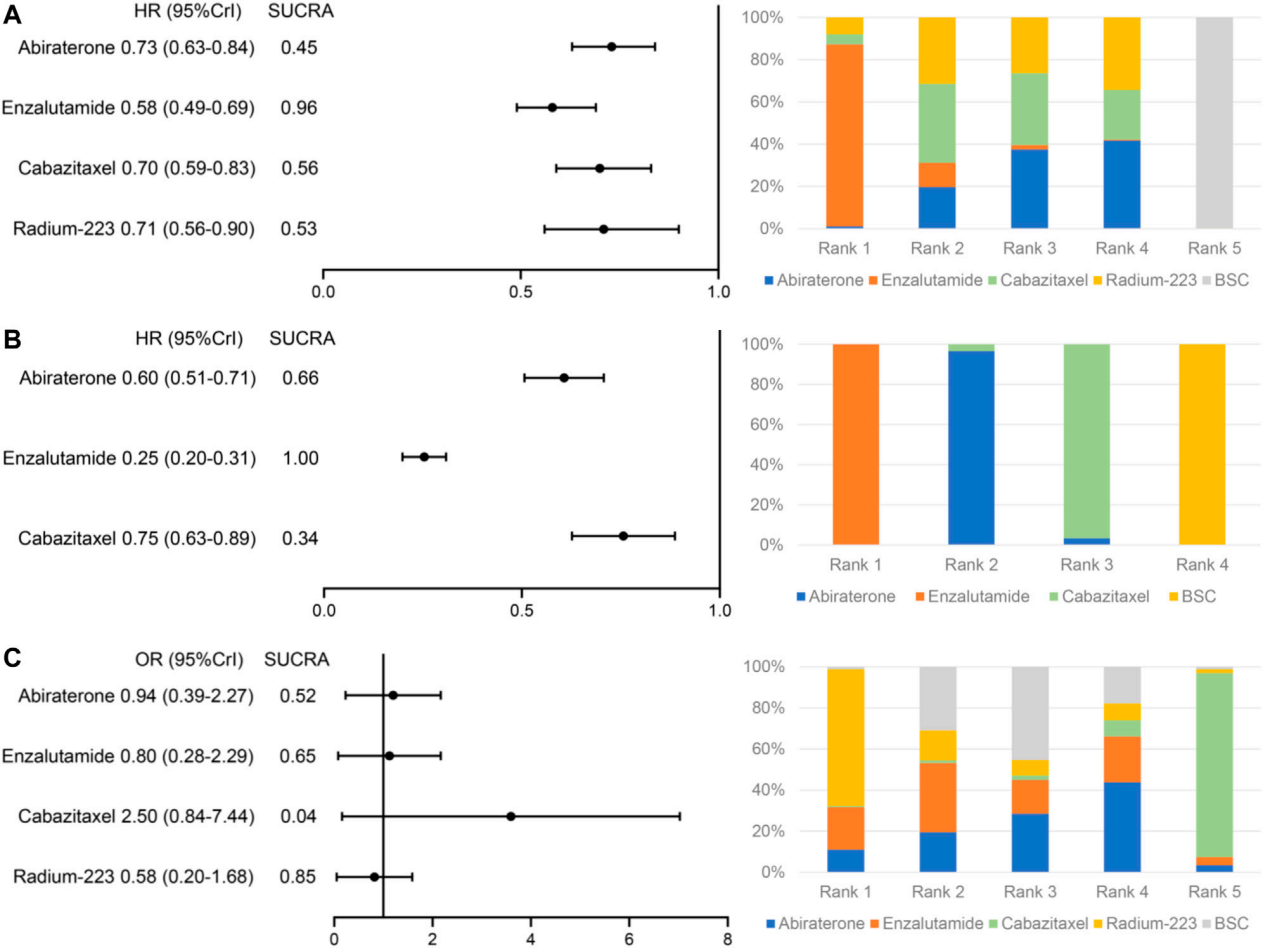
Relative effects of systemic treatments compared to best supportive care and treatment ranking on **(A)** overall survival, **(B)** biochemical progression-free survival, and **(C)** serious adverse events.

All the included studies reported outcomes of OS. Compared to BSC, abiraterone (HR = 0.73, 95% Crl: 0.63–0.84), enzalutamide (HR = 0.58, 95% Crl: 0.49–0.69), cabazitaxel (HR = 0.70, 95% Crl: 0.59–0.83), and radium-223 (HR = 0.71, 95% Crl: 0.56–0.90) showed significantly improved OS (Figure 3A). There was no significant heterogeneity (*I* ^*2*^ = 0). Furthermore, enzalutamide was associated with the highest probability of ranking first, followed by cabazitaxel, radium-223, and abiraterone, and the corresponding SUCRAs were 0.96, 0.56, 0.53, 0.45. The relative effect estimates for all pairwise treatment comparisons are summarized in Table 4.

**TABLE 4:** Relative effect estimates for all pairwise treatment comparisons.

Outcomes	Comparator	Intervention				
OS, HR (95%CrI)		Abiraterone				
	Enzalutamide	1.30 (1.06–1.59)	Enzalutamide			
	Cabazitaxel	1.04 (0.83–1.30)	0.83 (0.65–1.06)	Cabazitaxel		
	Radium-223	1.05 (0.78–1.41)	0.82 (0.61–1.10)	0.99 (0.73–1.34)	Radium-223	
	BSC	0.73 (0.63–0.84)	0.58 (0.49–0.69)	0.70 (0.59–0.83)	0.71 (0.56–0.90)	BSC
bPFS, HR (95%CrI)		Abiraterone				
	Enzalutamide	2.40 (1.80–3.20)	Enzalutamide			
	Cabazitaxel	0.80 (0.63–1.02)	0.33 (0.25–0.44)	Cabazitaxel		
	BSC	0.60 (0.51–0.71)	0.25 (0.20–0.31)	0.75 (0.63–0.89)	BSC	
SAE, OR (95%CrI)		Abiraterone				
	Enzalutamide	1.10 (0.29–4.20)	Enzalutamide			
	Cabazitaxel	0.39 (0.09–1.62)	0.33 (0.07–1.51)	Cabazitaxel		
	Radium-223	1.60 (0.39–6.56)	1.40 (0.31–6.32)	1.72 (0.59–5.01)	Radium-223	
	BSC	0.94 (0.39–2.27)	0.80 (0.28–2.29)	2.50 (0.84–7.44)	0.58 (0.20–1.68)	BSC

The bPFS data of patients with prior docetaxel were not available in the ALSYMPCA trial. Thus, radium-223 was excluded from the treatment comparisons with respect to bPFS. The network meta-analysis showed obvious improvements in bPFS of abiraterone (HR = 0.60, 95% Crl: 0.51–0.71), enzalutamide (HR = 0.25, 95% Crl: 0.20–0.31) and cabazitaxel (HR = 0.75, 95% Crl: 0.63–0.89) relative to BSC (Figure 3B). No significant heterogeneity was observed (*I* ^*2*^ = 20%). Additionally, as shown in Figure 3B; Table 4, enzalutamide was associated with superior bPFS outcomes compared to abiraterone (HR = 0.42, 95% Crl: 0.32–0.55) and cabazitaxel (HR = 0.33, 95% Crl: 0.25–0.44) with a 100% probability of being the best treatment. The corresponding SUCRAs were 1, 0.66, and 0.34.

**FIGURE 4:**
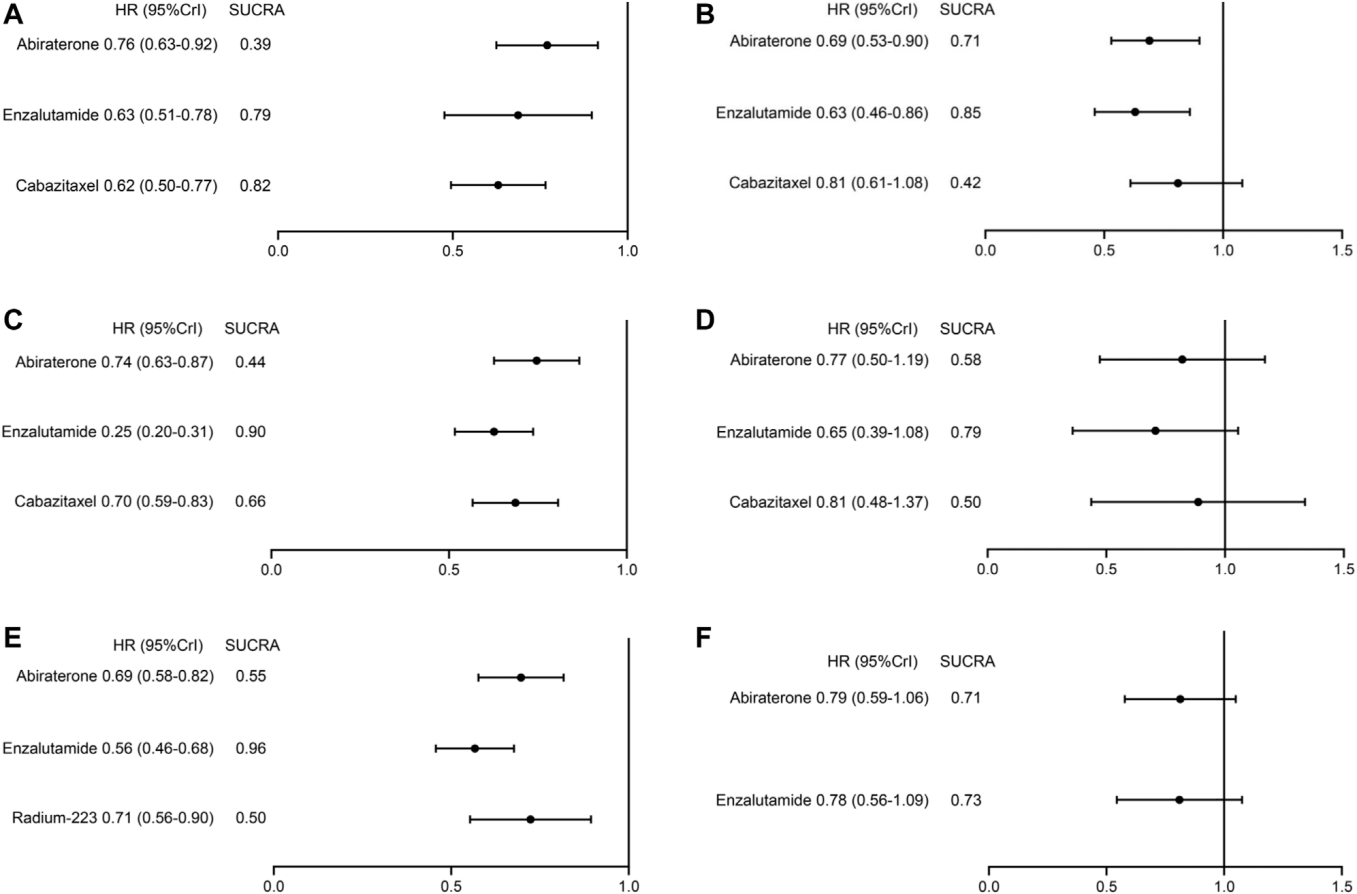
Treatment ranking and relative effects of systemic treatments from subgroup analyses on overall survival. **(A)** patient age≥65 years, **(B)** patient age<65 years, **(C)** patients with an ECOG score<2, **(D)** patients with a ECOG score≥2, **(E)** patients without visceral metastases, **(F)** patients with visceral metastases.

Subgroup analyses on OS were conducted according to patient baseline characteristics including age, ECOG score, and the presence of visceral metastasis. The results are summarized in Figure 4. Overall, patients with non-visceral metastasis or an ECOG score<2 were more likely to benefit from treatments. On the contrary, none of the treatments (abiraterone, enzalutamide, cabazitaxel) showed significantly improved survival compared to BSC in patients with visceral metastasis or a high ECOG score. In patients with younger age (<65 years), non-visceral metastasis, or an ECOG score<2, enzalutamide was associated with the highest probability of ranking first. However, in patients older than 65 years, cabazitaxel showed a superior ranking to other treatments.

### 3.4 Safety Outcomes

The data of SAEs were available in all the included studies. SAE outcomes of the COU-AA-301 and TROPIC trials were extracted from clinicaltrials.gov (NCT00638690 and NCT00417079). Treatments ordered from the safest to the least safe based on the risk of SAEs were radium-223 (OR = 0.58, 95% Crl: 0.20–1.68), enzalutamide (OR = 0.80, 95% Crl: 0.28–2.29), abiraterone (OR = 0.94, 95% Crl: 0.39–2.27) and cabazitaxel (OR = 2.50, 95% Crl: 0.84–7.44). The corresponding SUCRAs were 0.85, 0.65, 0.52, and 0.04 (Figure 3; Table 4).

## 4 Discussion

Currently, there is a lack of evidence regarding the preferred treatment for patients with mCRPC after docetaxel failure. In this study, we included five eligible RCTs and conducted a Bayesian network meta-analysis to comprehensively compare and rank four systemic treatments based on their efficacy and safety. All the drugs including abiraterone, enzalutamide, cabazitaxel, and radium-223 were associated with prolonged OS and bPFS relative to BSC. Among them, enzalutamide showed relatively superior survival benefits and higher ranking than others in the overall population and most of the subgroups. For safety, radium-223 was associated with the lowest risk of SAEs, enzalutamide and abiraterone with intermediate risk of SAEs, and cabazitaxel with the highest risk of SAEs.

Compared to the previous review (Fryzek et al., 2018), our study provides several novel findings. First, radium-223 was included in the network analysis. Approximately 90% of patients with mCRPC present with bone metastases (Tannock et al., 2004; Sun et al., 2016). Radium-223 could selectively bind to bone metastases and emit high-energy alpha particles to target areas (Bruland et al., 2006). In overall comparison, radium-223 was associated with prolonged OS. Considering that the ALSYMPCA trial excluded patients with visceral metastasis, we further performed subgroup analysis for patients without visceral metastasis. Consistently, radium-223 showed improved survival compared to BSC and similar efficacy with abiraterone and enzalutamide in this setting. Second, the prior indirect analysis used PFS as one of the efficacy outcomes, while the definitions of PFS were different cross trials (Fryzek et al., 2018). In this study, we compared the bPFS outcomes, which were measured more consistently in the included trials.

Moreover, patients with mCRPC are clinically heterogeneous, we performed subgroup analyses based on patient baseline characteristics to inform decision-making in specific populations. Across treatments, better efficacy was observed in patients with better performance status (ECOG<2) and in patients without visceral metastasis. Across patient subgroups, enzalutamide had the highest probability of being the best treatment for the most time. Patient age seemed to have little impact on the efficacy of enzalutamide and abiraterone, while older (≥65 years) patients might benefit more from cabazitaxel treatment. A recent study suggested abiraterone plus apalutamide combination therapy was associated with improved survival outcomes compared to abiraterone alone in older mCRPC patients who were chemotherapy-naïve (Saad et al., 2021). Thus, AR-targeted combination therapy may also be a potential option in post-chemotherapy setting. In terms of patients with higher ECOG scores, all treatments failed to yield significant survival improvements. It is possible that these patients may be less tolerant to AEs and have higher probability of treatment discontinuation (Honecker et al., 2018). For patients with visceral metastasis, neither abiraterone nor enzalutamide was associated with significantly prolonged survival relative to BSC, which was consistent with the situation in the chemotherapy-naive mCRPC setting (Beer et al., 2014; Ryan et al., 2015). Emerging evidence has suggested that variant histology subtypes and molecular aberrations in visceral metastasis were associated with treatment resistance (Pouessel et al., 2007; Akfirat et al., 2013). In the TROPIC trial, cabazitaxel was not analyzed in this subgroup, as patients were divided by measurable disease rather than visceral disease. However, a recent retrospective study demonstrated visceral metastasis was also an independent predictor for poor survival in patients treated with cabazitaxel (Kosaka et al., 2018). Furthermore, in the CARD trial, no difference in survival was observed between cabazitaxel and abiraterone/enzalutamide in heavily treated patients harboring visceral metastasis (de Wit et al., 2019). Thus, visceral metastasis remains a challenge in the treatment of mCRPC and future trials are needed to further address this issue.

In treatment selection, safety should also be taken sufficiently into consideration. Among the four treatments, radium-223 had the lowest risk of SAEs, followed by enzalutamide and abiraterone. Cabazitaxel was associated with the highest risk of SAEs. Specifically, hematological AEs were extremely common in patients with cabazitaxel. Combined with the survival outcomes, enzalutamide seemed to have the optimal efficacy and safety profile among the four systemic treatments.

Our findings should be interpreted with caution as this review is not devoid of limitations. First, the BSC for patients in the control arm varied across trials. Prednisone was used as BSC in most trials, while the TROPIC trial administered mitoxantrone plus prednisone as control. Although mitoxantrone plus prednisone showed comparative effectiveness with prednisone alone in mCRPC after docetaxel failure, the inconsistent comparators could still bias the results against cabazitaxel (Green et al., 2015). Second, with a follow-up duration of 12.9 months, median survival was not reached in either treatment arm in the Sun et al. (2016) trial. However, the relative effects and ranking probability of abiraterone remained nearly unchanged in the sensitivity analysis in which this trial was excluded (data not shown). Third, our results are limited by the potential bias of the TROPIC and ALSYMPCA trials, which also reflects the opportunities for future trials to improve study design. Finally, in the era of precision oncology, several biomarkers have emerged to predict treatment response, such as androgen receptor splice variant 7 (AR-V7) (Scher et al., 2018; Armstrong et al., 2019). A recent study showed that patients with nuclear-localized AR-V7 protein in circulating tumor cells might have longer survival if treated with taxane chemotherapy than AR signaling inhibitors. However, the relevant information was not feasible in the included trials.

## 5 Conclusion

This interactive network meta-analysis provides the best current evidence on the efficacy and safety profiles of multiple second-line treatments after docetaxel failure in patients with mCRPC. Our findings demonstrate that enzalutamide may provide optimal efficacy and a relatively low risk of SAEs. Cabazitaxel is also effective in post-docetaxel settings but associated with a high risk of SAEs. This study offers important implications for patients and clinicians. However, the results should be used with caution due to the inherent biases across the comparisons. Further head-to-head trials are needed to confirm our findings.

## Data Availability

The original contributions presented in the study are included in the article/supplementary material, further inquiries can be directed to the corresponding authors.
